# Squamous Cell Carcinoma Arising in a Squamous Odontogenic Tumor of the Maxilla: Case Report and Review of the Literature

**DOI:** 10.1007/s12105-024-01692-9

**Published:** 2024-10-24

**Authors:** Fawaz Alotaibi, Yousef Alshamrani, Harish Tummala, Abdulrahman Hesham, Leticia Ferreira Cabido, Mehrnaz Tahmasbi, John M. Wright

**Affiliations:** 1 Louisiana State University Health Sciences Center Shreveport, Shreveport, USA; 2 Texas A&M University, College Station, USA

**Keywords:** Squamous odontogenic tumor, Squamous cell carcinoma

## Abstract

Squamous odontogenic tumor (SOT) is an exceedingly rare, benign epithelial odontogenic tumor showing squamous differentiation. It is composed of variably sized and shaped islands of cytologically bland, mature squamous epithelium within a fibrous stroma. In this report, we present a rare transformation of a squamous odontogenic tumor (SOT) of the maxilla into a well-differentiated squamous cell carcinoma (SCC) with involvement of the pterygoid plates. To the best of our knowledge, only two cases of malignant transformation of SOT has been reported in the literature. Herein, we seek to report this extremely rare occurrence to raise awareness of oral and maxillofacial surgeons and pathologists of this unusual, but serious event and perform a literature review of squamous odontogenic tumors.

## Introduction

The squamous odontogenic tumor (SOT) is a benign, rare, slow growing, and locally infiltrative neoplasm arising from the cell rests of Malassez, gingival surface epithelium, or remnants of dental lamina [1, 2]. This entity was first identified by Pullon et al. in 1975 [2]. SOT has been recognized in the World Health Organization (WHO) Classification of Head and Neck Tumors in 2005, 2017, and most recently in 2022, as “a benign epithelial odontogenic tumor in which the tumor cells show terminal squamous differentiation” [3]. There has been a suggestion that NOTCH receptors and their ligands play a role in the cytodifferentiation of SOT [4]. In addition, a novel mutation of the Ameloblastin (AMBN) gene has been detected in a SOT, suggesting that this gene may play an important role in the tumorigenesis of this lesion [5]. SOTs are exceedingly rare with only 104 cases documented up until Chrcanovic et al. paper in 2018 [1] and less than 50 recognized cases by the WHO [3]. The age predilection of SOT is in the third through fifth decades of life and is typically diagnosed at a mean age of 34–38 years of age with no apparent sex predilection [1, 3, 6, 7]. The tumor typically presents as a solitary lesion; however, multifocal cases have been documented [8]. SOT typically affects erupting and vital teeth and often presents between the roots of teeth as a triangular-shaped radiolucency between diverging apices of adjacent roots [6, 9]. It affects the maxilla and mandible equally but shows a predilection for the anterior region of the maxilla and the posterior mandible [10]. An intraosseous variant and a peripheral variant have been described, with the latter being an extremely rare finding [6]. Typical treatment of a biopsy-proven squamous odontogenic tumor involves conservative surgical excision, enucleation and curettage with few documented cases of recurrence [1, 6]. With extensive disease, en bloc resection has been suggested as treatment modalities [1, 10]. So far in the literature, there have been only two cases reported malignant transformation of SOT [11]. Herein, we present the third case of malignant transformation of SOT which involved the maxilla of an adult male Table 1.

**Table 1:** Documented squamous odontogenic tumors with malignant transformation

Authors	Location	Age	Sex	Final pathology	Final treatment
Ide et al. [11]	Left posterior mandible	53 y/o	Male	Moderately differentiated intraosseous SCCa arising from SOT	Radical Resection of left posterior mandible
Pardhe et al. [12]	Right posterior mandible	69 y/o	Male	Well differentiated SCCA with islands of SOT	Referred for further surgical treatment
Alotaibi et al. 2024	Left posterior maxilla	64 y/o	Male	Well differentiated SCCa arising within SOT	Chemotherapy and radiation

## Case Report

**Fig. 1:**
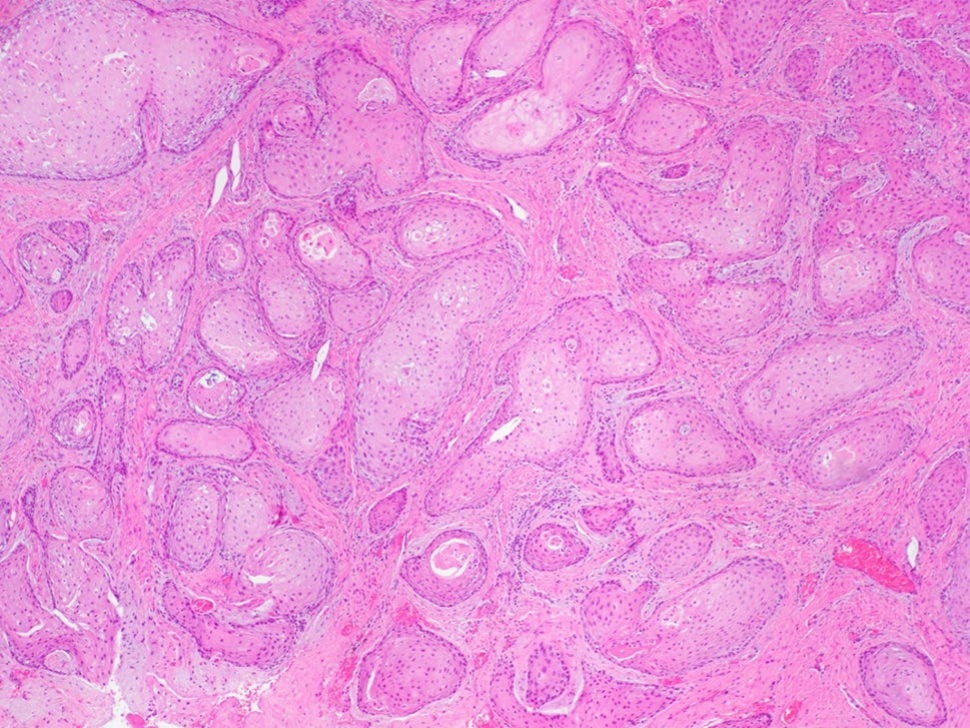
Initial biopsy of squamous odontogenic tumor showing multiple islands of terminally differentiated islands of squamous epithelium packed together like a “jig-saw puzzle.” H&E stain. Original magnification × 12

**Fig. 2:**
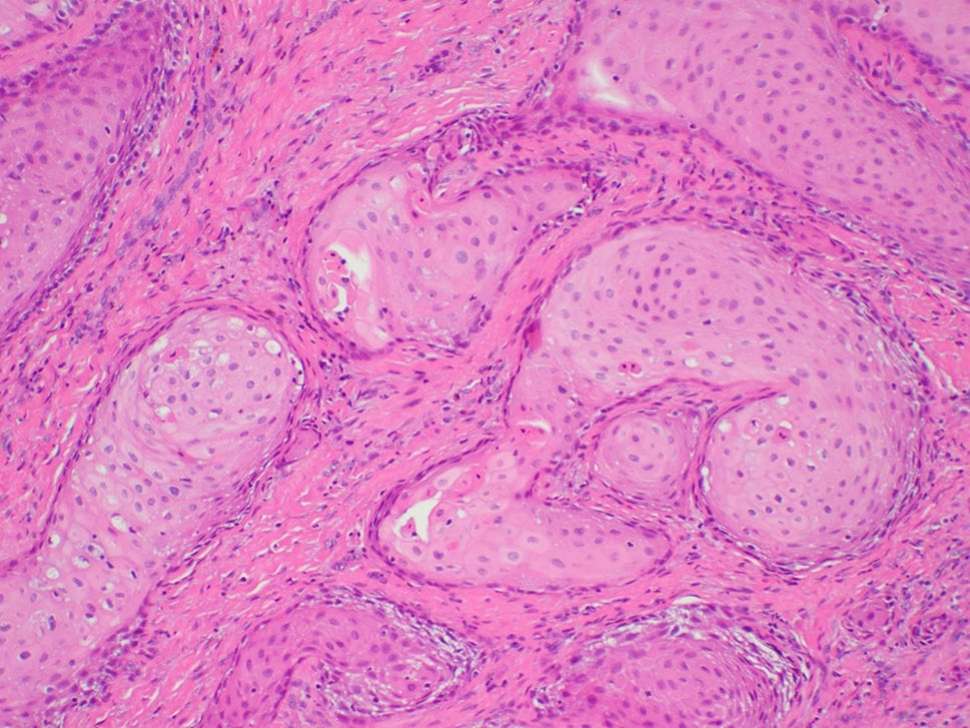
Initial biopsy showing well differentiated islands of squamous epithelium without peripheral palisading or cytologic atypia. H&E stain. Original magnification × 33

**Fig. 3:**
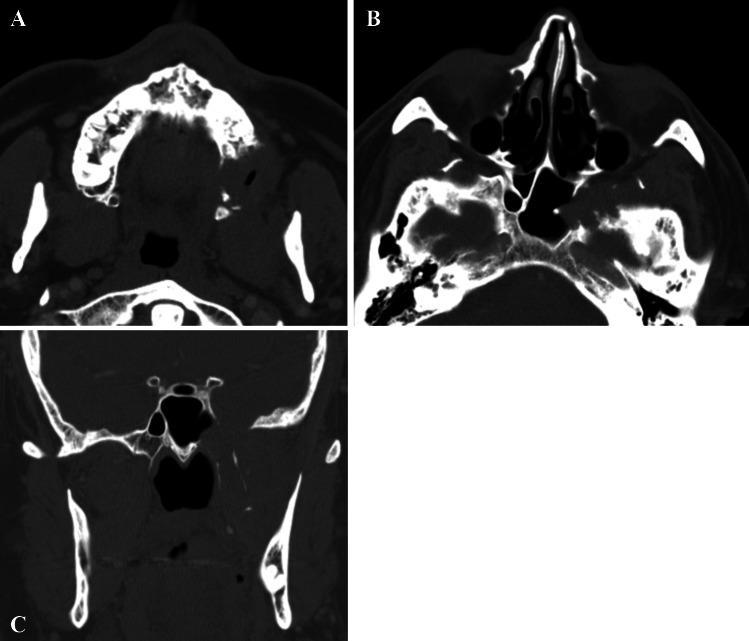
**A**–**C** MDCT bone window axial images (**A**, **B**) and coronal image (**C**). Depicting an ill-defined osteolytic lesion located on the posterior left side of the maxillary alveolar bone, sphenoid bone, and pterygoid plates

**Fig. 4:**
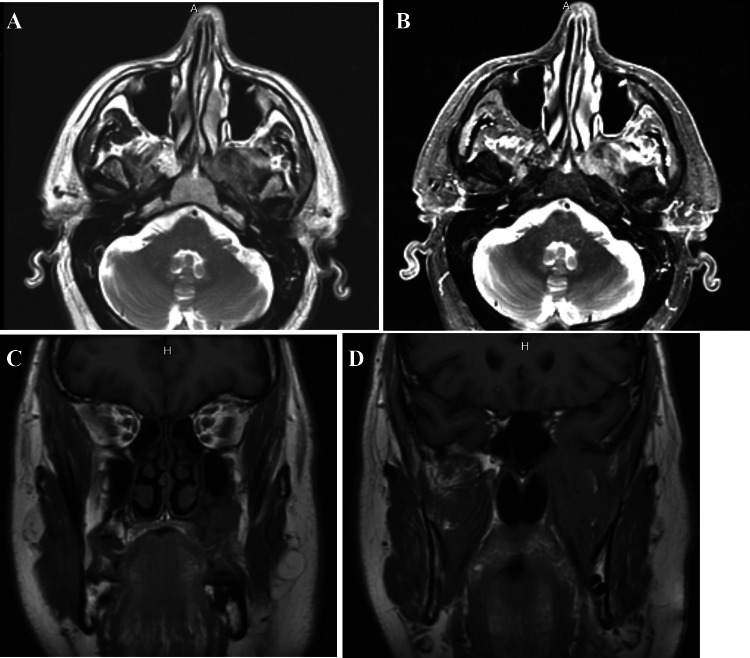
**A**–**D**: T1-weighted axial (**A**), T2-weighted axial (**B**), and T1-weighted coronal (**C**, **D**) MRI images. Indicating a soft tissue mass with hypointense signal intensity on T1-weighted and heterogeneous signal intensity on T2-weighted images, affecting the left posterior alveolar bone of the maxilla, the left infratemporal fossa, base of the skull, pterygopalatine fossa, and sphenopalatine foramen

**Fig. 5:**
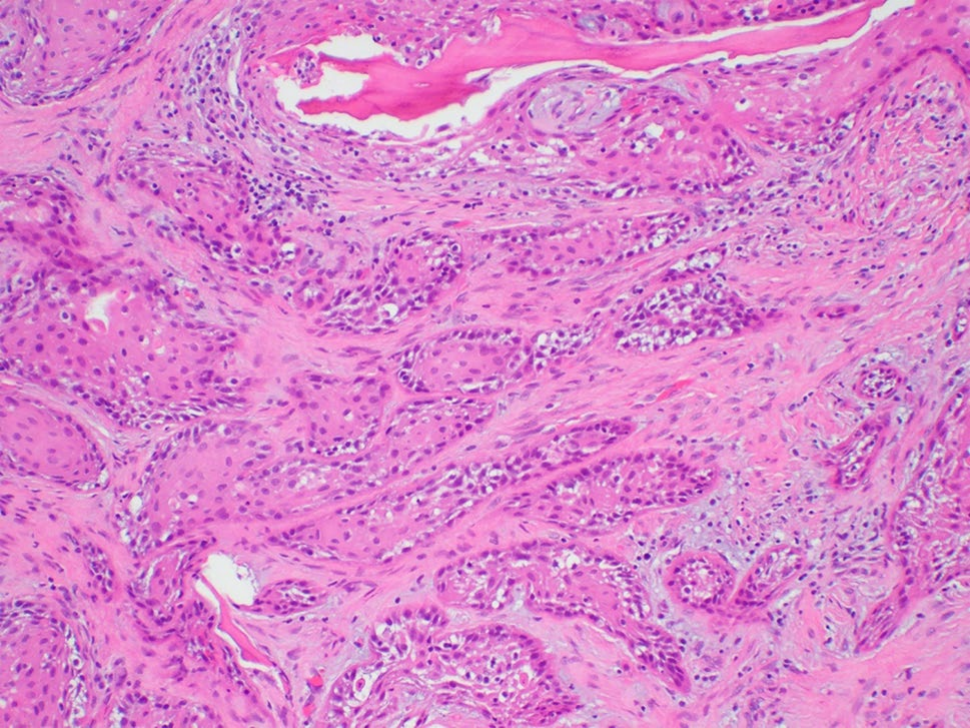
Area of squamous cell carcinoma where islands are more irregular and show significantly more cytologic atypia. H&E stain. Original magnification × 33

**Fig. 6:**
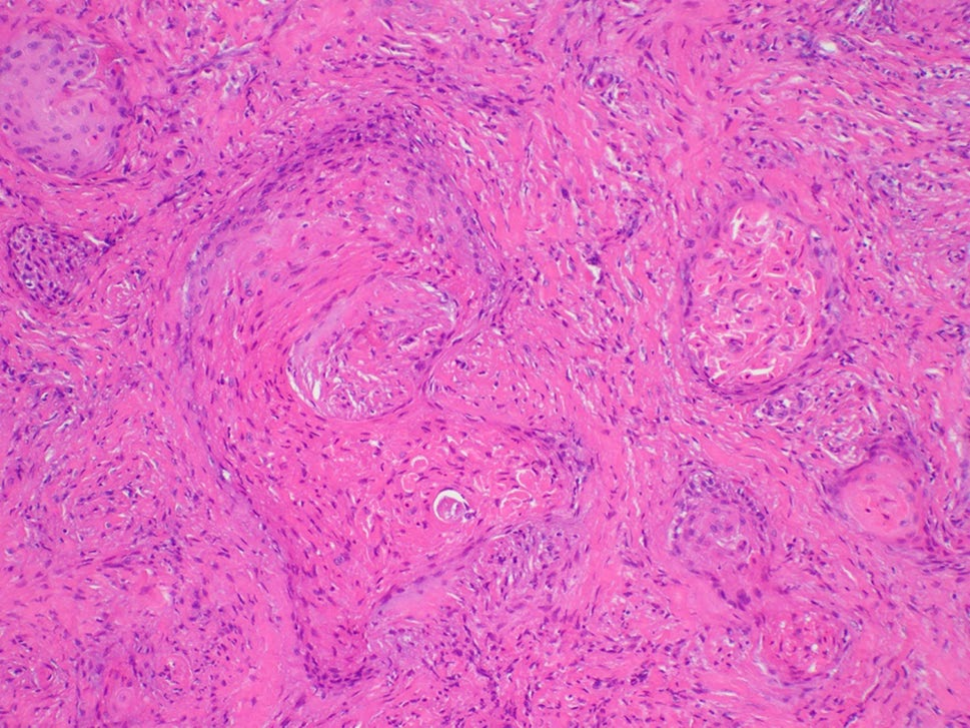
Area of squamous cell carcinoma with islands of tumor showing cytologic atypia and significant dyskeratosis. H&E stain. Original magnification × 66

**Fig. 7:**
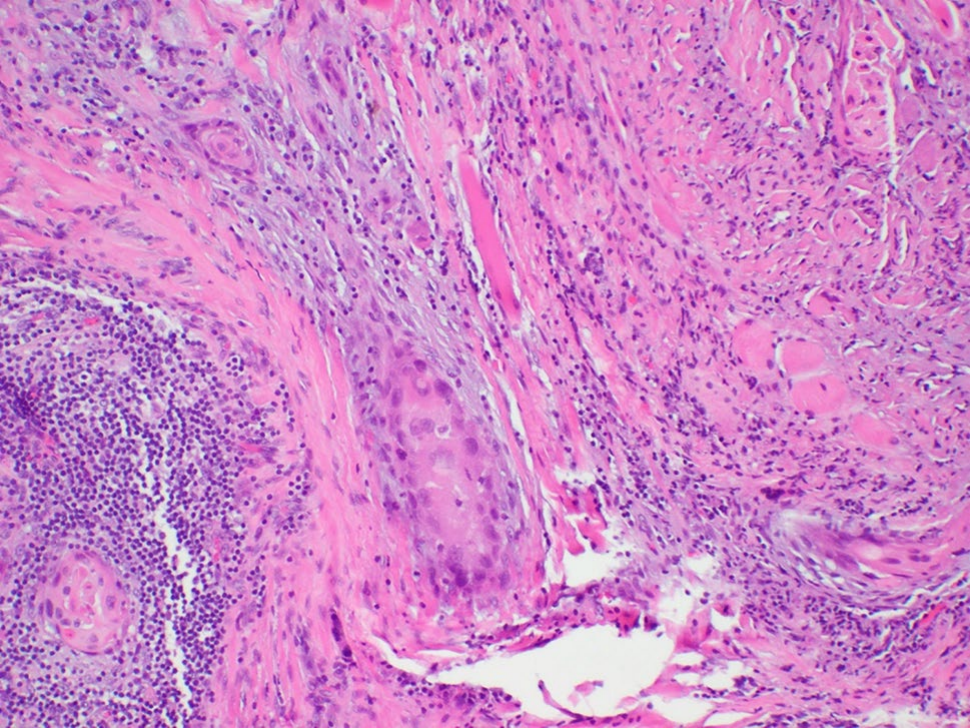
Area of muscle invasion by squamous cell carcinoma. H&E stain. Original magnification × 66

**Fig. 8:**
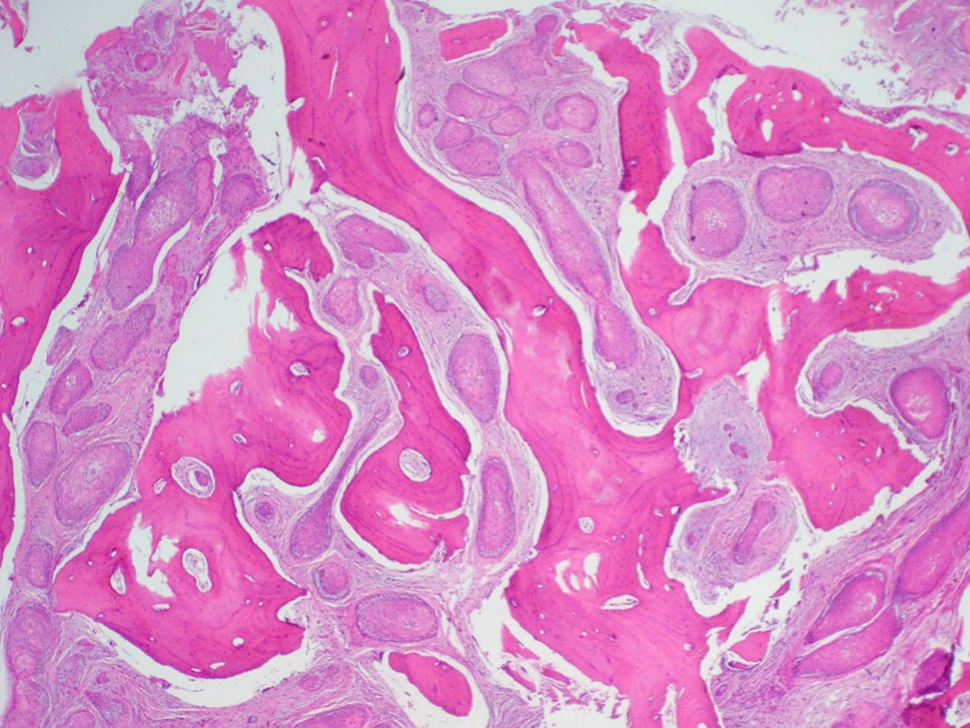
Area of bone invasion by squamous cell carcinoma. H&E stain. Original magnification × 12

**Fig. 9:**
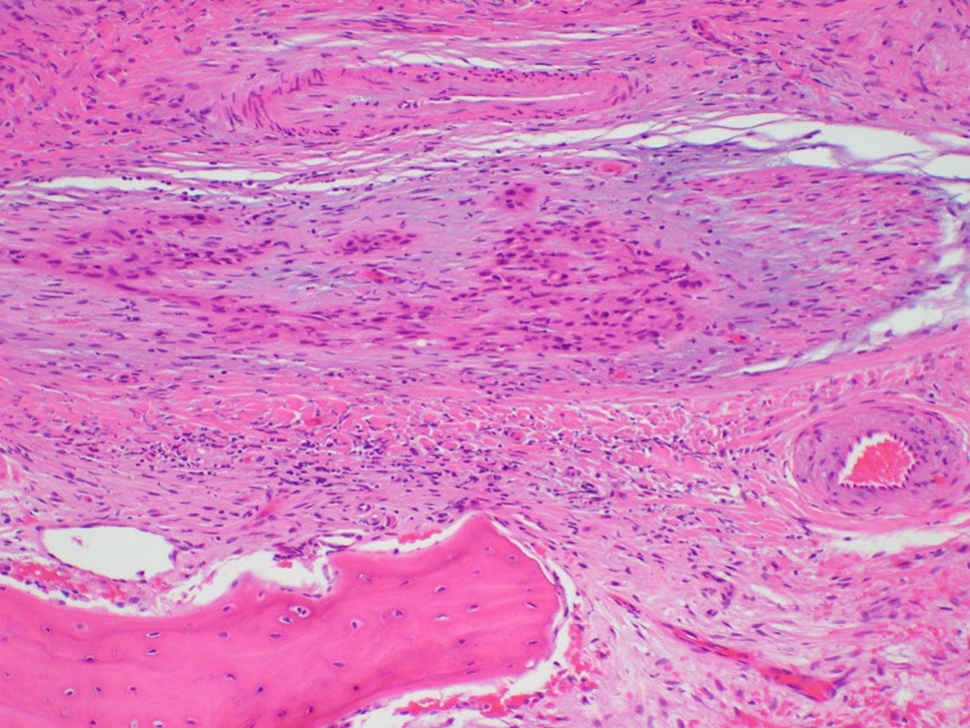
Areas of intraneural invasion by squamous cell carcinoma. H&E stain. Original magnification × 66

**Fig. 10:**
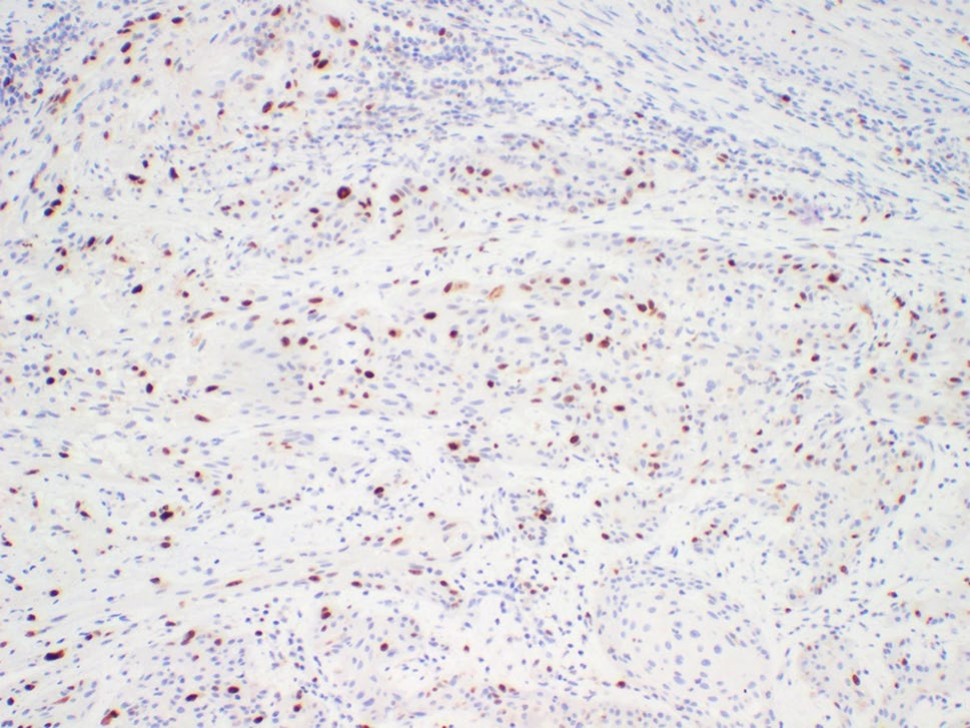
Ki-67 immunohistochemistry reaction showing increased nuclear reactivity in the areas of carcinoma. Original magnification × 33

**Fig. 11:**
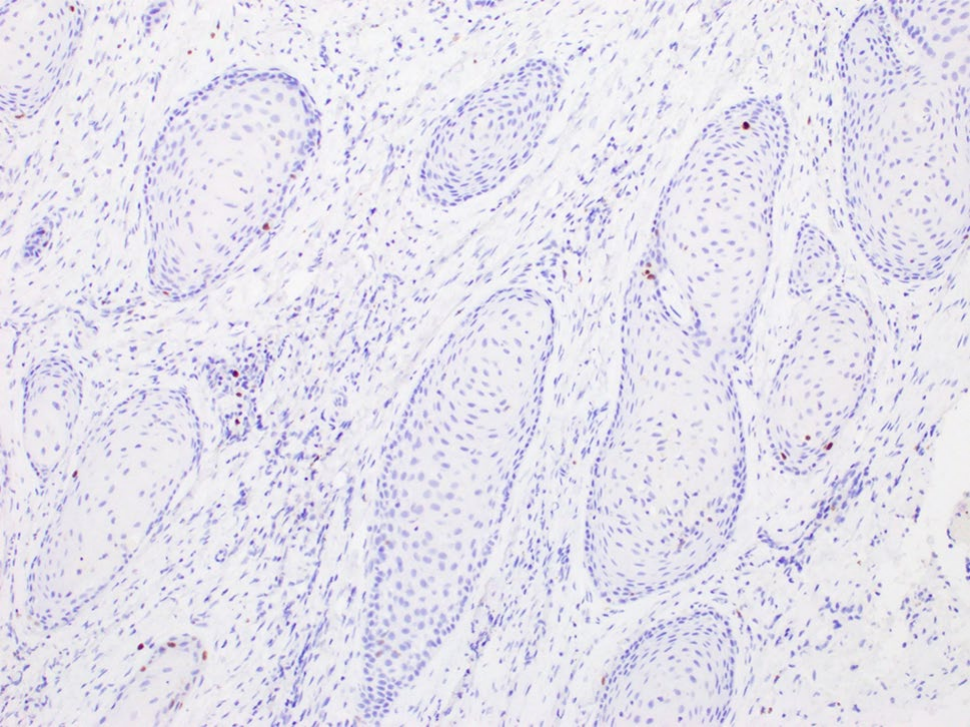
Ki-67 immunohistochemistry reaction showing markedly low nuclear reactivity in areas of the tumor containing only the benign squamous odontogenic tumor. Original magnification × 100

**Fig. 12:**
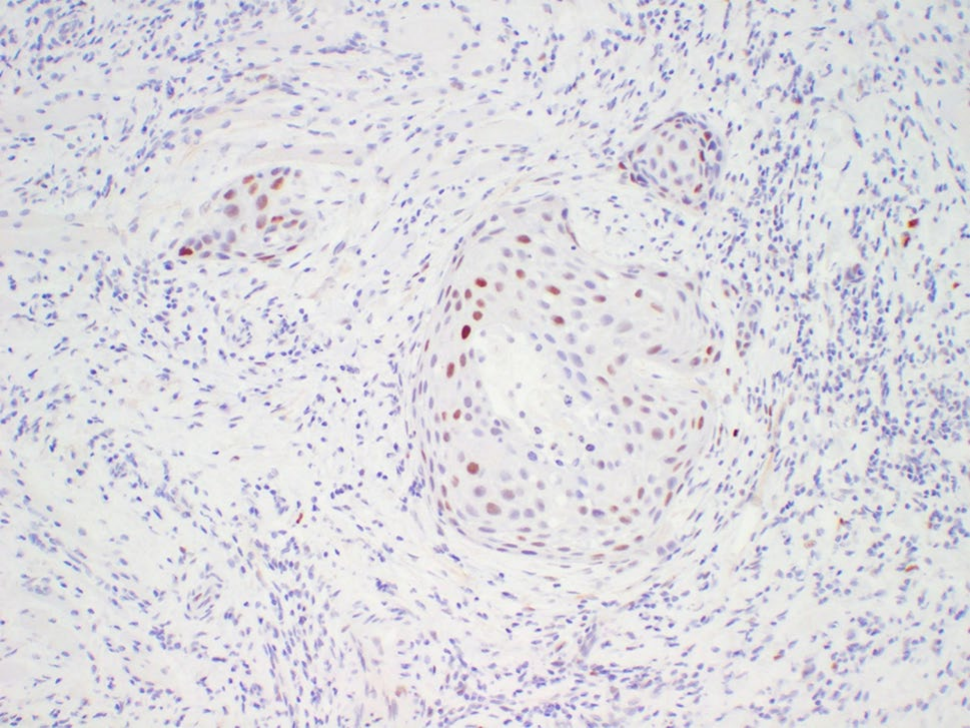
p53 immunohistochemistry reaction showing considerable nuclear uptake in areas of the carcinoma. Original magnification × 66

**Fig. 13:**
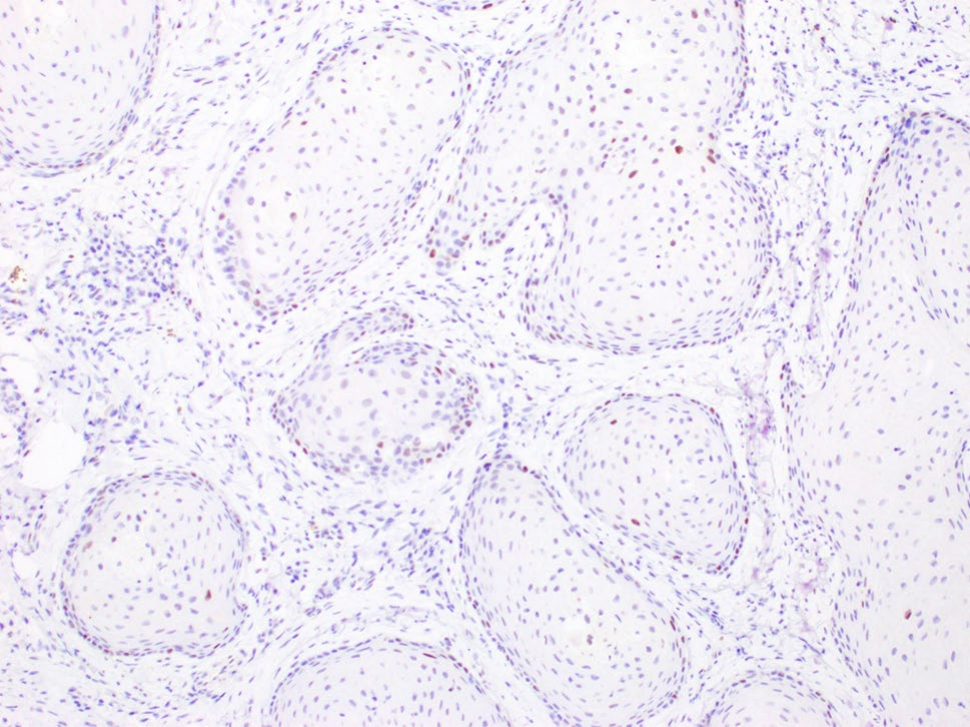
p53 immunohistochemistry reaction showing only weak, patchy nuclear uptake in areas of the benign squamous odontogenic tumor. Original magnification × 100

A 64-year-old male was referred to our clinic due to a non-healing extraction socket in the site of tooth #16. The patient underwent extraction of tooth #16 due to caries and pain by his dentist. Subsequently, the patient developed oroantral fistula. The patient had been suffering with persistent oroantral fistula and trismus after multiple procedures over the course of two years prior at another institution. An incisional biopsy was obtained and revealed multiple variably shaped islands of mature squamous epithelium tightly packed like a “jig-saw puzzle.” The peripheral cells were flattened and lacked ameloblastic differentiation. All cells were terminally differentiated and showed no cytologic atypia or mitoses. Very limited individual cell dyskeratosis was observed. A diagnosis of squamous odontogenic tumor was made (Figs. 1, 2). Our extraoral examination revealed that the patient had no vision changes, no palpable neck lymph nodes, intact trigeminal nerve, and no palpable swelling or bony expansion. Intraorally, the patient was noted to have an oro-antral fistula, non-healing, irritated mucosa, at the extraction site of tooth #16. A medical computerized tomography (CT) scan and a magnetic resonance imaging (MRI) were performed at that time. The CT scan showed an ill-defined osteolytic lesion on the posterior left side of the maxilla, with disruption of the buccal and palatal plates. The lesion involved the sites of the missing maxillary molars and the maxillary tuberosity. This lesion was accompanied by a soft tissue mass extending superiorly towards the base of the skull, involving the infratemporal fossa (Fig. 3). The lesion exhibited hypointense signal intensity on T1-weighted MRI images and relatively heterogeneous signal intensity on T2-weighted and STIR MRI images. These radiographic features were indicative of an aggressive lesion (Fig. 4). The patient was then taken to the operating room for surgical excision with left posterior maxillectomy, left coronoidectomy, and closure with buccal fat pad flap. The lesion along with the posterior maxilla, including the pterygoid plates, were removed and sent for microscopic examination. Histologically, the final specimen retained areas consistent with squamous odontogenic tumor. However, other areas showed smaller more angular islands of tumor with considerably more stroma in which the cells displayed cytologic atypia with some pleomorphism and nuclear hyperchromatism (Figs. 5, 6). There were areas of distinct muscle, intraneural and bone invasion (Figs. 7, 8, 9). A Ki-67 immunohistochemical stain was performed as a proliferation index and the areas of carcinoma were considerably more reactive (Fig. 10) than the more benign appearing areas (Fig. 11). Additionally, P53 immunohistochemistry was performed, and the areas of carcinoma were considerably more reactive (Fig. 12) than the benign areas of the tumor (Fig. 13). Given these findings, a diagnosis of well-differentiated squamous cell carcinoma arising in a squamous odontogenic tumor was established. Post operatively, the patient healed without any complications and started adjuvant chemoradiation therapy as recommended by our multidisciplinary tumor board.

## Discussion

There are only a limited number of reports in the literature of SOT which included appropriate radiographic, clinical, and histological documentation. According to Chrcanovic et al. in 2018, only 110 cases of central and peripheral SOTs reported in the literature met such criteria [1]. In contrast, in 2021, Upadhyaya et al. performed a literature review of SOTs including only cases with histologic photomicrographs depicting the histopathologic criteria described by the WHO and excluding SOTs associated with other tumors or cysts which resulted in only 50 acceptable cases of SOT [6]. Cases involving transformation of this lesion into squamous cell carcinoma are even more scarce. Two case reports have been published describing the transformation of SOT into SCC [11, 12]. In addition to that, Norris et al. reported transformation of SOT, however it lacks histological documentation of the proposed transformation of a mandibular SOT [11, 13]. The first well-documented transformation of a SOT into a SCC was reported twenty-five years ago by Ide et al. [11]. The tumor in that report presented as a small, well-defined radiolucency in the left posterior mandible, associated with an impacted tooth #17. The patient underwent an incisional biopsy at the time of extraction of #17, and the microscopic diagnosis identified it as SOT. Like our case, it was only after reviewing the excised enucleated tumor, that a diagnosis of SOT with scattered foci of SCC was rendered. Subsequently, their patient developed an extensive osteolytic lesion with destruction of the inferior cortex, which required radical treatment for SCC [11]. The original biopsy specimen in our case report was a cytologically bland presentation of SOT with no signs of cellular atypia or dyskeratosis, in contrast to our final excisional specimen which showed unequivocal cytological features of SCC. In 2007, Ruhin et al. described a maxillary lesion in a 9-year-old patient which was diagnosed as SOT on biopsy and recurred one year later after initial curettage [14]. The recurrent tumor showed significantly aggressive features, causing tumefaction of the maxilla and infiltration of the adjacent lip and nasal floor [14]. The recurrent specimen was diagnosed as “SOT in an aggressive form”. The photomicrographs of the recurrent tumor, however, depict islands of squamous epithelium showing dyskeratosis and cellular atypia, compatible with a well-differentiated squamous cell carcinoma [14]. Because there are no photomicrographs of the biopsy specimen in that report, it is difficult to determine if this case was also an example of malignant transformation of a SOT or if it was a well-differentiated squamous cell carcinoma from the beginning [14]. In the literature review of well-documented cases of SOT performed by Upadhyaya et al. 22% of the cases received aggressive treatment in the form of radical surgery, consisting of en bloc resection, hemimaxillectomy, or radical alveolectomy. According to the authors, those cases either presented as a multilocular radiolucency, presented as extensive or multifocal lesions, or exhibited recurrence [6]. Interestingly, many of these were located in the maxilla, suggesting that tumors in this site may have a slightly more aggressive biological behavior [6].

Histologically, SOT may be confused microscopically with acanthomatous ameloblastoma. Pullen et al. described the SOT to have islands of squamous epithelium with a similar morphological appearance to that of follicular ameloblastoma and was also compared to the squamous metaplasia seen in the acanthomatous ameloblastoma. Differences noted between the squamous metaplasia of acanthomatous ameloblastoma and SOT are the presence of keratin pearls in the former versus purely squamous appearing islands in the latter [2]. 

There has been a suggestion that NOTCH receptors and their ligands play a role in the cytodifferentiation of SOT [4]. In addition, a novel mutation of the Ameloblastin (AMBN) gene has been detected in a SOT, suggesting that this gene may play an important role in the tumorigenesis of this lesion [5]. The presence of chronic inflammation, exposure to carcinogens, genetic predisposition with accumulation of genetic alterations and viral infections has all been attributed as potential causes of tumor development [15]. In the setting of infection and chronic inflammation, leukocytes and phagocytes have been attributed to constantly causing DNA damage with generation of reactive oxygenated species and nitrogen species [16–19]. These cells are then exposed to the mutagens which can lead to transformation [15, 17, 19]. Our patient has had multiple prior surgical interventions to close the fistula and a long-standing chronic inflammation to the SOT cells. In the context of benign pathology, the mechanism above has been theorized to cause transformation of a benign entity to a malignant entity as documented by Jain et al. [20].

Another consideration in this case is sample bias leading to misrepresentation of the lesion which in essence would require a larger specimen to evaluate for evidence of malignant entity as provided by our final surgical specimen which was discussed by Bodner et al. [19]. SOT has been described in the literature as a lesion that is often misdiagnosed as variants of ameloblastoma, pseudoepitheliomatous hyperplasia or squamous cell carcinoma [6]. Ruskin et al. also described a mandibular lesion that the original microscopic diagnosis returned as a SOT [21]. The patient had a local recurrence which was then diagnosed as an acanthomatous ameloblastoma which on second review showed a primary intraosseous carcinoma [21]. Ide et al. demonstrated a final specimen showing a typical pattern of SOT with the presence of SCC in a restricted area with the carcinoma cells in ameloblastoma-like organization [11].

Treatment of SOT is typically enucleation and curettage with consideration for partial maxillectomy or marginal versus segmental mandibulectomy depending on the clinical aggressiveness of the lesion [1, 3, 6, 10]. In our case, we selected a posterior maxillectomy sacrificing the pterygoid plates due to the initial clinical presentation of the lesion. Buchner et al. have attributed bony erosion from SOT to pressure necrosis of the lesion versus neoplastic invasion [22]. However, after final pathological diagnosis, it becomes evident in concurrence with the radiographic evidence that the bony erosion in this case is most likely from the invasive SCC component of this transformed SOT. With a formal diagnosis of well differentiated squamous cell carcinoma, treatment must be directed as such. Standard treatment modalities involve radical resection with or without neck dissection, radiation, chemotherapy and/or immunotherapy [23, 24]. An important component of treating SCC is a formalized tumor board discussion to evaluate all options and formulate a consensus based on disease staging, patient performance status with consideration to morbidity of each modality. This patient was discussed at our institution’s multidisciplinary tumor board and the final recommendations are adjuvant chemotherapy and radiation due to the extent of the disease with a final staging of pT4bN0M0 squamous cell carcinoma.

## Conclusion

To the best of our knowledge, this is the third documented case of SCC arising in an SOT and it is the first one documented in the maxilla. The SOT is a rare entity and with SCC transformation in it is exceedingly rare. As evidenced by this case report, when assessing a SOT careful consideration of diagnosis must be given to include malignant transformation and malignant entities. Careful consideration must be given for appropriately sized specimens to avoid sampling bias and capture transformation to allow for appropriate treatment.

## Data Availability

No datasets were generated or analysed during the current study.
